# What are the core recommendations for gout management in first line and specialist care? Systematic review of clinical practice guidelines

**DOI:** 10.1186/s41927-023-00335-w

**Published:** 2023-06-15

**Authors:** Brooke Conley, Samantha Bunzli, Jonathan Bullen, Penny O’Brien, Jennifer Persaud, Tilini Gunatillake, Michelle M Dowsey, Peter F Choong, Mandana Nikpour, Rebecca Grainger, Ivan Lin

**Affiliations:** 1 Department of Surgery, The University of Melbourne, St Vincent’s Hospital Melbourne, Level 2, Clinical Sciences Building, 29 Regent St, Fitzroy, VIC 3065 Australia; 2 Department of Physiotherapy, The University of Melbourne, Melbourne, VIC Australia; 3 School of Health Sciences and Social Work, Griffith University, Brisbane, QLD Australia; 4 Physiotherapy Department, Royal Brisbane and Women’s Hospital, Brisbane, QLD Australia; 5 EnAble Institute, Curtin University, Perth, WA Australia; 6 Arthritis and Osteoporosis Western Australia, Perth, WA Australia; 7 Physiotherapy Department, Sir Charles Gairdner Hospital, Nedlands, WA Australia; 8 Departments of Medicine and Rheumatology, The University of Melbourne at St. Vincent’s Hospital, Melbourne, VIC Australia; 9 Department of Medicine, University of Otago Wellington, Wellington, New Zealand; 10 Te Whatu Ora Health New Zealand – Capital Coast and Hutt Valley, Wellington, New Zealand; 11 The University of Western Australia, Western Australian Centre for Rural Health, Geraldton, WA Australia; 12 Geraldton Regional Aboriginal Medical Service, Geraldton, WA Australia

**Keywords:** Evidence-based care, Gout, Practice guidelines, Evidence-based Medicine.

## Abstract

**Background:**

Gout is the most common inflammatory arthritis, increasing in prevalence and burden. Of the rheumatic diseases, gout is the best-understood and potentially most manageable condition. However, it frequently remains untreated or poorly managed. The purpose of this systematic review is to identify Clinical Practice Guidelines (CPG) regarding gout management, evaluate their quality, and to provide a synthesis of consistent recommendations in the high-quality CPGs.

**Methods:**

Gout management CPGs were eligible for inclusion if they were **(1)** written in English and published between January 2015-February 2022; focused on adults aged ≥ 18 years of age; and met the criteria of a CPG as defined by the Institute of Medicine; and **(2)** were rated as high quality on the Appraisal of Guidelines for Research and Evaluation (AGREE) II instrument. Gout CPGs were excluded if they required additional payment to access; only addressed recommendations for the system/organisation of care and did not include interventional management recommendations; and/or included other arthritic conditions. OvidSP MEDLINE, Cochrane, CINAHL, Embase and Physiotherapy Evidence Database (PEDro) and four online guideline repositories were searched.

**Results:**

Six CPGs were appraised as high quality and included in the synthesis. Clinical practice guidelines consistently recommended education, commencement of non-steroidal anti-inflammatories, colchicine or corticosteroids (unless contraindicated), and assessment of cardiovascular risk factors, renal function, and co-morbid conditions for acute gout management. Consistent recommendations for chronic gout management were urate lowering therapy (ULT) and continued prophylaxis recommended based on individual patient characteristics. Clinical practice guideline recommendations were inconsistent on when to initiate ULT and length of ULT, vitamin C intake, and use of pegloticase, fenofibrate and losartan.

**Conclusion:**

Management of acute gout was consistent across CPGs. Management of chronic gout was mostly consistent although there were inconsistent recommendations regarding ULT and other pharmacological therapies. This synthesis provides clear guidance that can assist health professionals to provide standardised, evidence-based gout care.

**Trial registration:**

The protocol for this review was registered with Open Science Framework (DOI 10.17605/OSF.IO/UB3Y7).

**Supplementary Information:**

The online version contains supplementary material available at 10.1186/s41927-023-00335-w.

## Background

Gout is the most common inflammatory arthritis, estimated to affect 1–4% of people worldwide [1]. The annual incidence of gout is 2.68 per 1000 persons and its prevalence is increasing with risk factors including dietary, genetic, and socioeconomic factors, presence of sustained hyperuricaemia or comorbid conditions [1–3]. Gout is a considerable burden for both people living with the condition and health care systems [4]. Per person, direct costs of USD $18,362 and indirect costs of USD $4,341 per annum are reported [5]. Cost and burden of gout are compounded by the high prevalence of comorbidities in people with gout, such as hypertension, diabetes mellitus, ischaemic heart disease, kidney disease and obesity [6].

Gout is both a metabolic urate accumulation disease and an autoinflammatory arthritic disease [7]. A gout flare is characterised by the onset of a painful, swollen, hot and red joint(s) [8]. After the first gout flare, recurrent flares can occur and gout may become tophaceous and/or erosive [9]. Optimal management of gout requires management of both gout attacks (autoinflammatory) and the metabolic pathway management (urate lowering treatment). Of the rheumatic diseases, gout is the best-understood and potentially most manageable condition, with complete control possible with safe, effective, and inexpensive pharmacological treatment when prescribed at the correct dosage and maintained long-term [2]. However gout frequently remains untreated or poorly managed [10]. Inconsistent recommendations across gout CPGs can result in confusion amongst health care practitioners, potentially contributing to sub-optimal gout management [11–13].

Clinical practice guidelines include recommendations intended to optimise patient care based on evidence and expert opinion [14]. Several CPGs have been published in recent years regarding the management of gout, albeit with variable quality and heterogenous recommendations [12, 15]. The purpose of this systematic review is to identify CPGs regarding gout management, evaluate their quality, and to provide a synthesis of consistent recommendations in the high-quality CPGs. By synthesizing these recommendations, healthcare providers such as general practitioners and rheumatologists will be supported in informed decision-making regarding gout management.

## Methods

### Search strategy and eligibility criteria

This systematic review was registered on the Open Science Framework (DOI 10.17605/OSF.IO/UB3Y7) and followed the Preferred Reporting Items for Systematic reviews guidelines [16]. A search strategy was developed for relevant terms related to CPGs (e.g., guideline*.mp. or Practice Guideline/ or Guideline/) and Gout. The full search strategy is available in Appendix 2. As this is part of a series of systematic reviews on arthritis management, search terms included other conditions and CPGs related to gout were identified from the articles yielded. The search was applied to the following databases: OvidSP MEDLINE, Cochrane, CINAHL, Embase and Physiotherapy Evidence Database (PEDro). A second search was conducted of four guideline repositories: Guidelines International Network, National Health and Medical Research Council, Effective Health Care from Agency for Health Care Research and Quality, and the National Institute for Health and Care Excellence. The search included CPGs published between January 2015 to February 14th, 2022 to reflect up-to-date research evidence (see Table 1). The list of included CPGs was reviewed by two rheumatologists who are clinician-researchers (MN and RG), to identify if any CPGs were missing to their knowledge.

**Table 1:** Clinical Practice Guidelines (CPGs) selection criteria

Inclusion criteria • Published between January 2015 –February 14th, 2022. • For the interventional management of gout. • For adults (people aged ≥ 18 years). • Published in the English language or in which a complete English language version is available. • Developed using a systematic process that is a guideline based on a systematic review of the literature and developed by an expert, multidisciplinary panel [14]. • Represents an original body of work i.e., not solely an adaptation or systematic review of existing guidelines.
Exclusion criteria • Does not include interventional management recommendations. • Includes other arthritic conditions. • **Only** addresses recommendations for the system/organisation of care. • Required additional payment to access.

### Protocol changes

The original protocol excluded CPGs that addressed one treatment modality only e.g., medication prescribing. However, to improve comprehensiveness of the review, CPGs addressing single interventions were included as they were considered important for gout management. This decision to widen the scope was made during the study selection phase of the review. The timeframe proposed in our original search strategy was between January 2015 and December 2020 but was extended to include published CPGs up until February 14th 2022.

### Study selection

Search results from the databases were aggregated in Endnote™ and duplicate records removed. Records were imported to the Covidence software program (Veritas Health Innovation, Melbourne, Australia. Available at www.covidence.org). Titles and abstracts were screened independently by two reviewers (BC and TG or IL) and assessed for eligibility according to criteria. Full texts articles were then screened, and final inclusion of articles was agreed on by consensus between the two reviewers or where necessary, consultation with a third reviewer (SB).

### Data appraisal: Quality assessment of guidelines

Clinical practice guidelines were assessed for quality using the Appraisal of Guidelines for Research and Evaluation (AGREE) II instrument [17]. The AGREE II is an internationally validated, widely used tool to assess the quality of CPGs in any disease area [17, 18]. Seven members of the research team undertook the online AGREE II practice exercise and were provided with the AGREE II user manual [17, 19]. Clinical practice guidelines were independently appraised by pairs of the reviewers (BC, JB, JP, PO, SB, TG). The AGREE II instrument consists of 23 individual items grouped in six domains: scope and purpose, stakeholder involvement, rigour of development, clarity of presentation, applicability, and editorial independence. Consistent with the AGREE II manual, each item was independently rated by two reviewers to increase the reliability of the assessment using a 7-point Likert scale from 1 (strongly disagree) to 7 (strongly agree) [17]. Domain scores were calculated, using the following formula: (Obtained score - minimum possible score / maximum possible score - minimum possible score) [17]. The AGREE II developers do not provide uniform criterion for overall quality and recommend research teams determine criteria based on their own circumstances [17]. Authors determined a cut-off score equal to or greater than 60% of the maximum possible score in three domains we believed were most important for validity. These were: rigour of development (domain 3), editorial independence (domain 6) and stakeholder involvement (domain 2). The three domains of interest are consistent with a previous review of CPGs in musculoskeletal pain management, and a value of 60% for ‘high quality’ is similar to other reviews [20–22].

### Inter-rater agreement

Domain percentages and overall assessment rating (%) for each reviewer were independently calculated. Our criteria for acceptable inter-rater agreement were domain percentages and overall scores that were less than or equal to 20% difference between reviewers (BC, JB, JP, PO, SB, TG) as intraclass coefficient values of 80 or above are considered either excellent or almost perfect [23, 24]. In instances where there was a variation of greater than 20% between scores, reviewers met to discuss the ratings and a rating was determined by consensus, involving a third reviewer if consensus was not achieved. Fifteen domains required consensus across six reviewers (BC, JB, JP, PO, SB, TG).

### Data extraction

Data were independently extracted by the first author (BC) using a purpose-designed Excel spreadsheet, adapted from a previous musculoskeletal review [21]. This comprised of CPG characteristics (e.g., title, country of publication), methodology, guideline topic target users (Appendix 4). Recommendations were extracted from CPGs and ranked as either ‘should do’, ‘could do’, ‘do not do’ or ‘uncertain’ (Appendix 3). Recommendation ratings were consistent with language used in the CPGs and a grading system of a previous musculoskeletal systematic review of CPGs [21]. While CPGs used variable language, their recommendations and quality of evidence were based on the same criteria GRADE [25–28], Oxford Centre for Evidence-Based Medicine standards [25, 29, 30] or an adaption of these tools [31]. In the CPGs a ‘should do’ meant that the recommendation was based on a high level of evidence and benefit unequivocal and a strong consensus from the development group. A ‘could do’ meant that consistent evidence from multiple lesser quality studies or one high quality study and where benefits outweigh harms and/or based on strong consensus with the development group. A ‘do not’ recommendation was based on either high- or low-quality studies where the harms outweigh the benefits. Whilst an ‘uncertain’ recommendation was stated if only poor-quality evidence was available, and the development group couldn’t recommend for or against. Three authors (SB, MN, RG) independently checked tables and interpretation of recommendations, any discrepancies were identified and resolved by discussion between the authors and rechecking data against the original citation.

### Narrative summary

The narrative summary was developed initially by one author (BC) and then reviewed and refined by four authors (SB, IL, MN and RG). The summary included a description of the number of CPGs that reported on an intervention, what the recommendations involved and highlighted areas where recommendations were consistently similar in their details or where there were inconsistencies (Appendix 7). Recommendations that were considered consistent between CPGs, were described as ‘consensus’ (see Appendix 1 Process of defining consensus between CPGs on individual recommendations).

## Results

### Data extraction

**Fig. 1:**
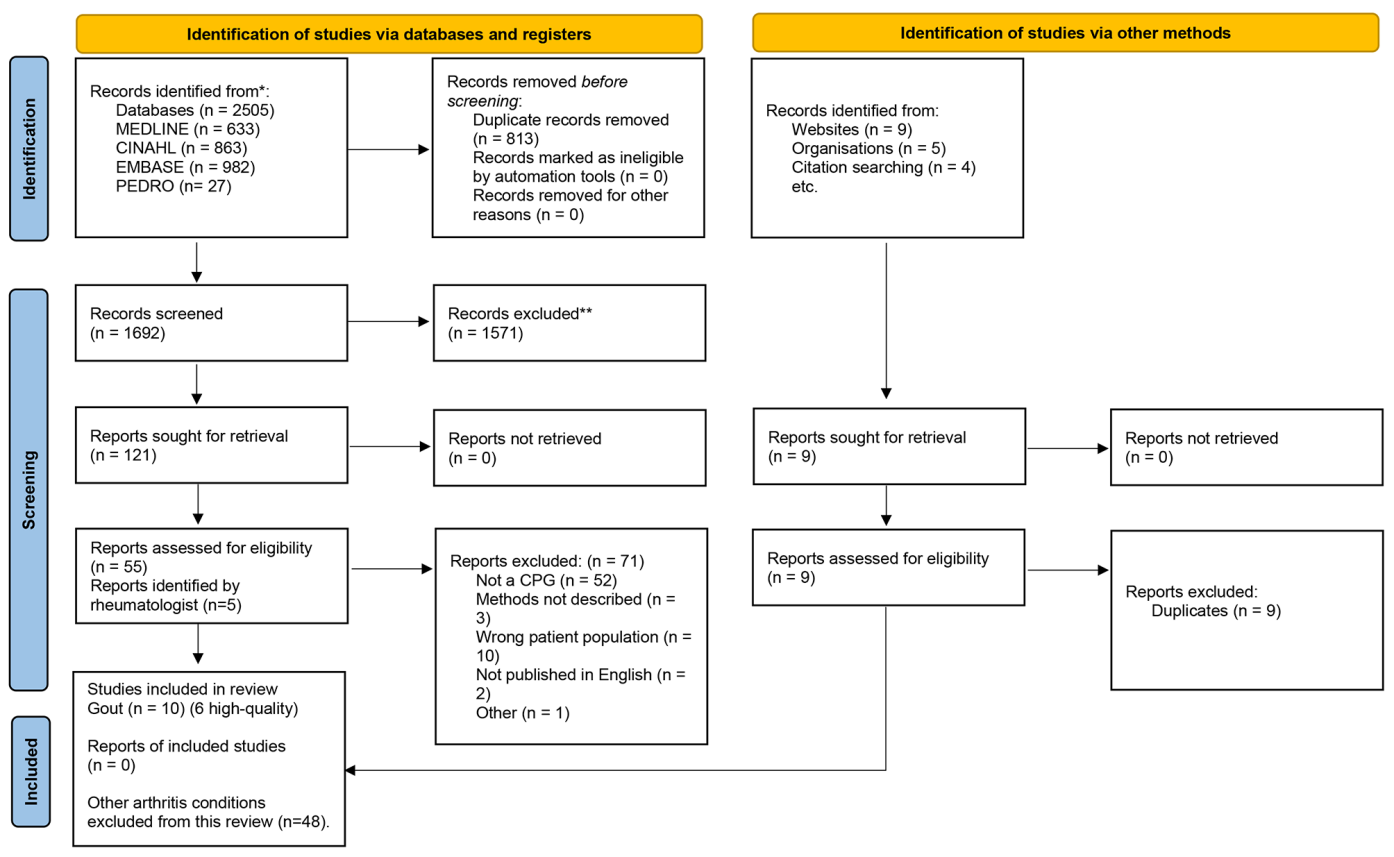
PRISMA 2020 flow diagram for new systematic reviews which included searches of databases, registers and other sources

Ten CPGs were identified. Six CPGs met the eligibility criteria [25–27, 29, 31, 32] and four were rated low quality on AGREE-II and excluded [33–36] (Fig. 1). All six high quality CPGs were developed by medical/professional societies, two societies were based in the United States of America [26, 27], three in Europe [25, 29, 32] and one in the United Kingdom [31]. Target users included health professionals [25–27, 29, 31, 32], people with gout [25, 26, 29, 31, 32] and their families [32], policy makers and those responsible for commissioning care [29, 31] (Appendix 4).

### Quality of CPGs

The AGREE II scores for each CPG are provided in Appendix 6. AGREE II results for included CPGs are provided in Appendix 6.1 with CPGs rated low quality on AGREE II and excluded presented in Appendix 6.2. Quality was assessed across six domains: scope and purpose (range: 67-94%), stakeholder involvement (range: 50-92%), rigor of development (range: 68-82%), clarity of presentation (range: 78-94%), applicability (range: 21-92%), and editorial independence (range: 25-100%). The mean (SD) AGREE II scores for each item, domain and overall scores across all guidelines are displayed in Appendix 5. The mean overall scores for all CPGs were 77% (SD = 12.4). The domain with the lowest mean score was ‘applicability’ (45%, SD = 24.8), and the highest mean score was for ‘clarity and presentation’ ​​(89%, SD = 6.8).

## Consensus recommendations (Fig. 2; Appendix 7)

**Fig. 2:**
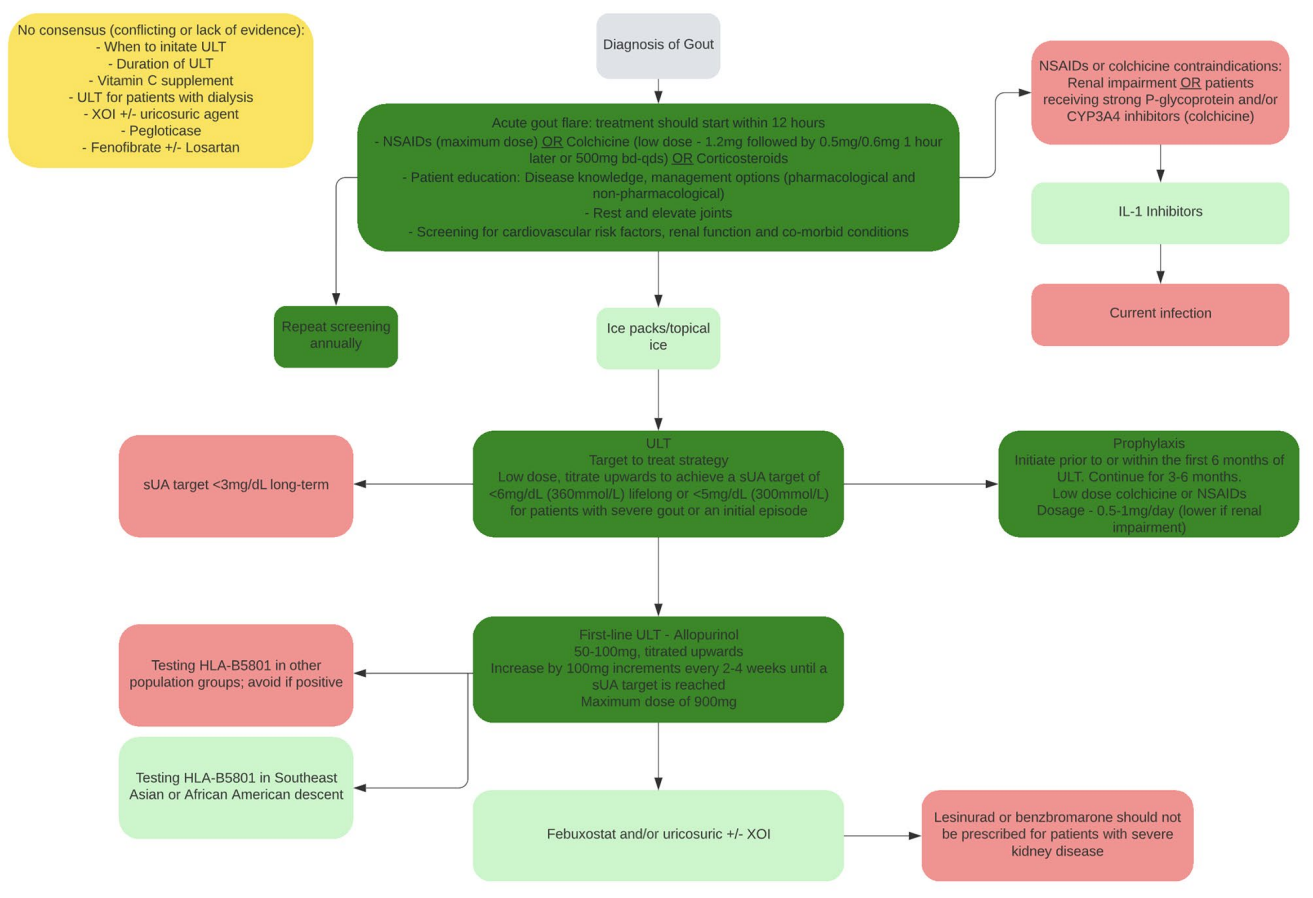
Gout management from synthesis of six CPG’s: **Key:** Dark green: Should do consensus recommendations. Light green: Could do consensus recommendations. Red: Do not do consensus recommendations or contraindicated. Yellow: No consensus recommendations. **Abbreviations**: sUA – serum uric acid; IL-1 – Interleukin-1; ULT – urate-lowering therapy; XOI – xanthine oxidase inhibitor.

## Treatment of gout flare

### Recommendations with ‘Should do’ consensus

The following recommendations were strongly recommended by two or more CPGs: non-steroidal anti-inflammatories (NSAIDs), colchicine and/or corticosteroids should be commenced as first-line medications [25–27, 29, 31, 32]. Patients should be provided with education on non-pharmacological and pharmacological management options and advice on elevating and resting the affected joint(s) [25–27, 29, 31]. Education should also include dietary advice and ULT [25, 27, 29, 31], weight loss for patients who are overweight or obese [25, 26, 29, 31], exercise advice for all patients, and (if applicable) smoking cessation [29]. Clinical practice guidelines did not report how to implement education topics specifically [25, 26, 29, 31]. Screening for cardiovascular risk factors (e.g., coronary heart disease, heart failure, stroke, peripheral arterial disease, renal impairment, obesity, hyperlipidaemia, hypertension, diabetes mellitus, and smoking) should be conducted and repeated annually [25, 29, 31]. Prophylaxis should be recommended prior to or within the first six months of initiating ULT and continued for at least 3–6 months with colchicine being the preferred treatment at a dosage of 0.5-1 mg/day followed by NSAIDs, or COX-2 inhibitors and low-dose glucocorticoids if the other options are contraindicated, not tolerated, or ineffective [25, 26, 29]. Patients with renal impairment should receive a reduced dose of prophylaxis [25].

### Recommendations with ‘Could do’ consensus

The following recommendations were conditionally recommended by two or more CPGs, or equally conditionally and strongly recommended: Cold therapies e.g. ice packs, could be used in combination with other evidence-based therapies [26, 31]. Interleukin-1 (IL-1) inhibitors can be considered for people with acute gout or who have frequent flares, who have contraindications or have not responded adequately to standard treatment of colchicine, NSAIDs and corticosteroids [25, 26, 29, 31].

### Recommendations with ‘Do not do’ consensus

The following recommendations were recommended against by two or more CPGs: IL-1 inhibitors should be avoided if the patient has a current infection [25]. NSAIDs should not be recommended for people with gout and severe renal impairment, who are experiencing an acute gouty attack [25, 29]. Similarly, colchicine should be avoided for patients with renal impairment or for patients who are receiving strong P-glycoprotein and/or CYP3A4 inhibitors such as cyclosporin or clarithromycin [25, 29, 31].

## Chronic gout management

### Recommendations with ‘Should do’ consensus

The following recommendations were strongly recommended by two or more CPGs: Serum uric acid (sUA) levels should be monitored and ULT should be based on a treat-to-target strategy and adjusted to achieve a sUA target < 6 mg/dL (360 mmol/L) lifelong or sUA < 5 mg/dL (300mmol/L) for patients with severe or tophaceous gout [25, 29, 32] with one CPG recommending this target can be considered for all patients with gout, based on expert opinion only [31]. Allopurinol should be recommended as a first-line ULT starting at 50-100 mg daily (no greater than 100 mg) and lower in patients with chronic kidney disease [25, 26, 29, 31, 32]. Allopurinol dose should be increased by 100 mg increments every 2–4 weeks or 4 weeks until sUA target is reached, with a maximum daily dose of 900 mg [25, 29, 31, 32].

### Recommendations with ‘Could do’ consensus

The following recommendations were conditionally recommended by two or more CPGs, or equally conditionally and strongly recommended: Febuxostat can be used as an alternative second-line xanthine oxidase inhibitor (XOI) for patients who have renal impairment and/or chronic kidney disease, where allopurinol is contraindicated or has not been effective in achieving the therapeutic sUA target [26, 29, 31, 32]. A low dose should be prescribed initially of 80 mg-<100 mg daily, increasing the dosage after 4 weeks to 120 mg daily, if necessary to achieve therapeutic target and lower for patients with chronic kidney disease [26, 31]. Uricosuric agents either in combination with a XOI or as monotherapy can be considered for patients who have a poor response, are intolerant or have an adverse reaction to XOI’s [29, 31, 32].

IL-1 inhibitors can be considered if a patient has contraindications or hasn’t responded to standard treatments for inflammation of gout such as: colchicine, NSAIDs and corticosteroids [25, 26, 29, 31]. If diuretics are being used to treat hypertension and the hypertension is controlled, other anti-hypertensive agents should be considered instead [25, 26, 31].

### Recommendations with ‘Do not do’ consensus

The following recommendations were recommended against by two or more CPGs: A sUA level < 3 mg/dL should be avoided long-term, due to the possibility of adverse effects that may be associated with a very low sUA [29, 31]. Allopurinol should be avoided in patients who have the HLA-B*5801 allele [32]. Uricosuric agents, lesinurad or benzbromarone should not be prescribed for patients with severe kidney disease [32]. Lesinurad should be avoided if a patient has experienced a vascular event in the last 12 months [32]. Measuring urinary uric acid and alkalinizing urine while using uricosuric treatment was recommended against, based on expert opinion [26]. The guideline development group concluded that uricosuric use is infrequent, there is a lack of evidence to support alkalinizing agents for patients with gout and challenges with regular testing such as, inaccurate results from diet [26]. IL-1 inhibitors should not be considered if the patient has a current infection [25]. Patients who are not of Southeast Asian descent (e.g., Han Chinese, Korean, Thai) or African American should not be tested routinely for HLA–B*5801 [26, 32].

### Recommendations with no consensus

Inconsistent recommendations were reported for when to initiate ULT, duration of ULT, vitamin C intake, pegloticase, fenofibrate and losartan [25, 26, 29, 31, 32]. Clinical practice guidelines varied on when to initiate ULT, ranging from at their first presentation, following diagnosis [25, 29, 31] to after a flare has settled [31], taking into consideration particular criteria [25, 26, 29, 31] (For full details, see Appendix 7). Duration of ULT varied from short term < 12 months [27] to indefinitely [26]. There were conflicting recommendations regarding the use of vitamin C with one CPG conditionally recommending for [31] and one against the use in managing gout [26], fenofibrate and losartan were recommended against [26, 31] although, one CPG conditionally recommended for their combined use in In treating comorbid hypertension or dyslipidaemia in people with gout [31]. Consensus recommendations are presented in Fig. 2.

## Discussion

Six of the ten CPGs identified were assessed as high quality on the AGREE II instrument. Quality scores were higher than in previous reviews [12, 15]. This may be due to the inclusion of more recent CPGs that are of higher quality, or because different criteria to define quality were used. The AGREE II domain ‘Applicability’ was frequently rated lower than other domains, suggesting developers paid limited attention on how CPGs are translated into practice.

There are consistent recommendations for acute gout management which are patient education, medication to treat inflammation (NSAIDs, colchicine, or corticosteroids) and screening to identify comorbid conditions such as cardiovascular and renal diseases. These anti-inflammatory medications report a similar high-level of efficacy and choice of treatment should be based on the presence of comorbidities and patient preference [37, 38]. Clinicians can be confident that by following these recommendations, they are offering high quality care, as these findings align with recent gout management reviews and have been supported for over two decades [2, 7, 37, 39].

Clinical practice guidelines recommended clinicians take on an educative role with patients around pharmacological management and lifestyle modifications such as, weight loss and dietary changes. However, implementation of education into practice remains an issue. People with gout report rarely receiving clear guidance on these topics possibly due to a lack of detail and clarity of these recommendations [40, 41]. The CPGs included in this review grouped dietary recommendations together, leading to a lack of clarity in the strength of recommendations for individual dietary choices. Similarly, CPGs recommended clinicians provide advice on other non-pharmacological interventions such as rest/elevation of affected joints, exercise and smoking cessation, although reported insufficient detail on how to apply these interventions in practice [25, 29, 31]. Future CPGs should provide further guidance on these educational topics and look to separate dietary recommendations based on their level of evidence. Individualised dietary advice should consider personal, social and cultural factors including comorbid health conditions, preferences and availability or access to food [42]. Clinicians, particularly general practitioners, have reported a lack of time in consultations as a barrier to effectively educating patients with gout [43]. Involvement of a multi-disciplinary team approach to care, can help address time concerns for limiting education and has been shown to be effective in lowering sUA levels [44–46]. In conjunction with face to face education, providing patients with informational materials such as written materials or web-based resources with content tailored to the patient, may improve uptake of behaviour change [45, 47]. Patient adherence to medical advice remains an issue. Providing clear, jargon-free instructions, at a level that can be understood by the patient and using pictures where appropriate, can facilitate uptake of education and advice [48]. Furthermore, development of therapeutic relationships and equipping patients with the skills and motivation to be adherent with their treatment is needed, is needed, as evidenced by the success of a nurse-led intervention for gout [6, 49, 50].

Urate lowering remains the mainstay of the long-term management of gout with a lack of adherence being recognised as the main cause of gout management failure [2, 51]. Adherence to ULT is low, varying from 20 to 70% [52]. This may be due to misconceptions about what gout is and how best to manage it such as: patients believing that gout is an acute disease which does not require ongoing management; concern about ULT side effects; and/or lack of confidence in ULT effectiveness due to experiencing a gout flare following initiating ULT [2, 43, 47, 53]. Clinical practice guidelines are very clear that education on gout medical management is critical to address a patients’ understanding of optimal management. Clinicians should explain the importance of taking ULT regularly and continually to prevent gout attacks and discuss possible side effects such as the risk of flares once commencing ULT [29, 31, 45, 54]. While it is important to educate people about dietary and lifestyle modifications, on their own these modifications do not lead to significant urate reduction, therefore education about the pharmacological management of gout is essential [4, 7, 37].

Clinician level barriers to effective medical management include a lack of referrals between primary and specialist’s care, knowledge gaps, conflicting recommendations from CPGs and similar misconceptions to patients such as: general practitioners treating gout as an acute condition only [12, 15, 43, 55]. Appropriate training, development of CPG consensus statements, clearly defined roles amongst healthcare practitioners and strengthening of networks between health professionals can help to address barriers [56]. While CPG can address care of individual people with gout, they do not usually address how to optimise health systems in the delivery of that care. In New Zealand multi-component interventions to reduce barriers to prescribing of ULT and enhance access to ULT have been successful in improving quality of gout care [49]. In the United Kingdom, nurse-led care gout care has been shown to increase the proportion of patients reaching target-serum urate and is cost effective [50]. It seems likely that addressing health systems and care delivery will be integral to achieving better health outcomes for people with gout.

Clinical practice guidelines were consistent in recommending ULT (preferably allopurinol) and prophylaxis for managing chronic gout. Allopurinol was recommended at a low dose of 50-100 mg daily (no greater than 100 mg) and titrated upwards [25, 26, 29, 31]. Research has shown that approximately 400mgs daily or above is often needed achieve sUA target [37]. Sub-optimal use of allopurinol is common, ULT is often underutilised by clinicians, or when it is initiated is often prescribed at a fixed dose, therefore underdosing [11, 54, 57]. Under-dosing may be due to fear of kidney damage, which has not been supported by evidence or allopurinol hypersensitivity, a rare yet highly fatal adverse reaction [4, 58–60]. Clinicians should be aware of potential side effects and prescribe ULT in accordance with CPGs guidance, titrating dose as required.

Clinical practice guidelines were inconsistent in their recommendation of when to commence ULT, varying from immediately following diagnosis, to only being indicated in certain clinical scenarios, based on an individual patients’ circumstances [25, 26, 29, 31]. This discrepancy could be due to no internationally agreed upon criteria, and this decision of when to commence ULT being based on the individual patients’ circumstances. One CPG did not provide a recommendation on this topic, and reported no clear guidance on ULT in general, sUA target and sUA monitoring [27, 61]. Clinical practice guidelines are divided as to whether it can or cannot be considered whilst the patient is experiencing a gout flare. Historically, it was thought that starting ULT during a gout flare could worsen or prolong the flare, however this has since been questioned [6]. Similarly, heterogeneity existed between CPGs of how long to continue ULT, with one CPG recommending to continue indefinitely and another recommending shorter durations (< 12 months) due to a lack of research investigating the long-term benefits of ULT for people with single or infrequent gout (< 2 attacks per year) [26, 27]. The American College of Physicians CPG recommendation to avoid long-term ULT use has been heavily criticised for its lack of clear, detailed guidance and for ignoring key ULT considerations such as the presence of tophi [61]. Differences in recommendations have been attributed to their interpretation of the literature by the development group, which included physicians, and reflects the common belief amongst the medical community that gout is an intermittent condition that does not require ongoing management [61]. Despite this recommendation, it is widely accepted that effective gout management requires long-term adherence to ULT, to achieve an optimal sUA target [54]. If CPGs report varying recommendations on when to commence and how long to continue ULT for, this could lead to either delayed commencement and/or failure to prescribe long-term ULT, contributing to the poor long-term management reported in gout patients.

### Further areas of research

Further research is needed to understand why gout CPGs differ in their recommendations and to determine recommendations on the current areas with no consensus: when to initiate ULT and length of ULT, vitamin C intake, pegloticase, fenofibrate and losartan. While developing high-quality CPGs is important, this alone is insufficient to improve health outcomes for people with gout [62]. Further research should focus on implementation strategies to encourage uptake of CPGs recommendations in clinical practice.

### Strengths and Limitations

Strengths of this systematic review include the use of AGREE II tool as a systematic approach to synthesis [17], and selection of a high-level quality cut-off value. Additionally, we involved a multi-disciplinary team, including rheumatologists (RG, MN), physiotherapists (IL, SB, JP, BC), an Aboriginal health researcher (JB), orthopaedic surgeon (PC), nurse and epidemiologist (MD) and social scientists (TG, PO). The review was conducted according to the published protocol, with only two minor amendments to expand the inclusion criteria so that CPGs on single aspects of gout management could be included and to update the search strategy. The AGREE II instrument reflects methodological processes, not necessarily content, and scores may reflect reporting rather than methodological quality. The research team defined high quality as 60% in the three domains of interest (domain 2, 3 and 6), similar to other reviews [21, 22]. Grading of interventions and consensus recommendations (e.g., ‘should do’, ‘could do’, ‘do not do’ or ‘unsure’ recommendations) were based on the language used in CPGs. However, CPGs differ in their use of language and recommendations may be based on expert opinion without strong evidence to support this decision. To improve confidence in our interpretations, consensus statements were developed by three authors (BC, SB and IL) and reviewed by the expert clinicians (RG and MN).

## Conclusions

This synthesis of current high-quality CPGs provides guidance for health care providers, on recommended gout care. Recommendations from six CPGs were that acute gout flare management should include anti-inflammatories, education, screening, and rest/elevation of the affected joints. Established gout should be managed with ULT and continued prophylaxis to prevent further gout flare and manage tophaceous and erosive gout. CPGs disagreed on when to initiate ULT and length of ULT, vitamin C intake, pegloticase, fenofibrate and losartan. This synthesis of recommendations is relevant to healthcare providers and can be implemented in clinical practice to standardise high-quality care and optimise patient outcomes.

## Electronic supplementary material

Below is the link to the electronic supplementary material.

**Supplementary Material 1.** Appendix 1.

**Supplementary Material 2.** Appendix 2.

**Supplementary Material 3.** Appendix 3.

**Supplementary Material 4.** Appendix 4.

**Supplementary Material 5.** Appendix 5.

**Supplementary Material 6.** Appendix 6.

**Supplementary Material 7.** Appendix 7.

## Data Availability

All data generated or analysed during this study are included in this published article [and its supplementary information files].

## Abbreviations

AGREE: Appraisal of Guidelines for Research and Evaluation
CPGs: clinical practice guidelines
GP: general practitioner
NSAIDs: non-steroidal anti-inflammatory
sUA: serum uric acid
IL-1: Interleukin-1
ULT: urate-lowering therapy
XOI: xanthine oxidase inhibitor
