# Supplementary material for: What are the core recommendations for gout management in first line and specialist care? Systematic review of clinical practice guidelines
Source: BMC Rheumatol. 2023 Jun 15;7:15. doi: 10.1186/s41927-023-00335-w (PMC10268528; doi:10.1186/s41927-023-00335-w)
Supplement: Supplementary file 1 — Supplementary Material 1. Appendix 1. [file 41927_2023_335_MOESM1_ESM.docx]

**Appendix 1. Defining consensus among CPGs regarding individual recommendations**
