# Supplementary material for: What are the core recommendations for gout management in first line and specialist care? Systematic review of clinical practice guidelines
Source: BMC Rheumatol. 2023 Jun 15;7:15. doi: 10.1186/s41927-023-00335-w (PMC10268528; doi:10.1186/s41927-023-00335-w)
Supplement: Supplementary file 3 — Supplementary Material 3. Appendix 3. [file 41927_2023_335_MOESM3_ESM.docx]

**Appendix 3. Classification of Gout recommendations**

|  | **BSR [31]** | **ACP [27]** | **EULAR [25]** | **ISR [29]** | **ACR [26]** | **SER [32]** |
| --- | --- | --- | --- | --- | --- | --- |
| **Non-pharmacological management** | | | | | | |
| Education (diet, exercise, weight management, ULT, rest, elevation, cool environment) | ✓✓ | ✓✓ | ✓✓ | ✓✓ | ✓ |  |
| Adequate water intake (>2L) | ✓✓ |  |  |  |  |  |
| Vitamin C | ✓ |  |  |  | O |  |
| Nurse involvement |  |  |  |  |  | ✓✓ |
| **Urate-lowering therapies** | | | | | | ? Patients on dialysis  ✓ Referred to unit with clinical experience |
| Initiating ULT | ✓✓ All patients especially recurring attacks; tophi; chronic; joint damage; renal impairment; a history of urolithiasis; diuretic therapy use; primary gout starting at a young age.  ✓ Delayed commencement |  | ✓✓ All patients especially recurrent flares; tophi; urate arthropathy and/or renal stones; young age (<40 years); very high sUA level; and/or comorbidities | ✓✓ Patients with recurrent flares; tophi; urate arthropathy and/or renal stones; very high sUA level; and/or comorbidities | ✓✓(≥ 1 subcutaneous tophi, radiographic damage, Gout flare >2/year)  ✓ (Infrequent flares <2/year, first flare and CKD stage >3, sUA >9 mg/dl, or urolithiasis)  O (First flare, asymptomatic hyperuricemia (sUA >6.8 mg/dl with no prior gout flares or subcutaneous tophi)  ✓ During a flare | ✓✓ ULT w XOI |
| Aim and monitoring sUA level combined | ✓✓ ≤300 mmol/l initial ✓ ≤360 mmol/l |  | ✓✓ 360 mmol/L (<6 mg/dL) ✓ 300 mmol/L (<5 mg/dL) severe gout O <3 mg/dL long term. | ✓✓ 360 mmol/L (<6 mg/dL) ✓✓ 300 mmol/L (<5 mg/dL) severe gout | ✓✓ 360 mmol/L (<6 mg/dL) | ✓ 300 mmol/L (<5 mg/dL)  ✓✓ Same for CKD |
| Dosage of ULT (Titration to reach sUA target) |  |  | ✓✓ |  | ✓✓ | ✓✓ |
| Length of ULT therapy |  | ✓ Short-term O Long-term |  |  | ✓ |  |
| **Acute treatment** | | | | | | |
| NSAID | ✓✓ | ✓✓ | ✓✓  O Severe renal impairment | ✓✓ O (Severe renal impairment) | ✓✓ | ✓✓ |
| Colchicine | ✓✓ | ✓✓ | ✓✓ O Colchicine if receiving strong P-glycoprotein and/or CYP3A4 inhibitors O Severe renal impairment | ✓✓ O Severe renal impairment | ✓✓ |  |
| Corticosteroids | ✓ | ✓✓ | ✓✓ | ✓✓ | ✓✓ | ✓✓ |
| **Prevention** | | | | | | |
| Prophylaxis |  |  | ✓✓ | ✓✓ | ✓✓ |  |
| Xanthine Inhibitor |  |  |  |  | ✓✓ CKD stage >3  ✓ switching first XOI to an alternate XOI | ✓✓ CKD  ✓✓ XOI and uricosuric agents |
| Allopurinol | ✓✓ |  | ✓✓ ✓ Renal impairment | ✓✓ ✓ Renal impairment | ✓✓ All patients including those with CKD stage >3  ✓ Allergy - allopurinol desensitization | ✓✓ All patients including those with CKD  O HLA-B*58 allele  ✓✓ CV event |
| Febuxostat | ✓ |  |  | ✓ Renal impairment | ✓✓ ✓ Switching ULT if history of CVD or a new CV event | ✓ First-line severe gout  ✓✓ CKD  ✓ High CV risk, no history of CV event – consider risk/benefits |
| Uricosuric agents | ✓ |  |  | ✓ | O Checking urinary uric acid and alkalinizing urine | ✓✓  Lesinurad:  ✓✓ Montherapy or + XOI Before benzbromarone  O Severe kidney disease  O CV event past 12 months  Benzbromarone  ✓ Poor  response to treatment, adverse reactions to XOI or CVD or CKD  O Severe kidney disease |
| Pegloticase |  |  | ✓ Severe, debilitating, chronic tophaceous gout, poor QoL, failed to achieve sUA target |  | O First-line therapy and patients who have failed to achieve the sUA target; infrequent gout flares; no tophi.  ✓✓ Failed to achieve sUA target; frequent gout flares; non-resolving subcutaneous tophi. | ✓ Refractory gout or no other treatment option  ✓✓ Severe kidney disease or who do not tolerate well other treatment options |
| Probenecid |  |  |  |  | ✓ |  |
| Fenofibrate +/- Losartan | O Primary ULT ✓ Hypertension or dyslipidaemia |  |  |  | O |  |
| IL-1 inhibitors | ✓ |  | ✓  O current infection | ✓ | ✓ |  |
| Antihypertensive medications | ✓ |  | ✓ |  | ✓ |  |
| Stopping aspirin |  |  |  |  | O |  |
| Two medications with the same mechanism of action |  |  |  |  |  | O |
| Medical vs Surgical |  |  |  | ✓✓ Medical intervention  ✓ Surgical mx in selected cases e.g. nerve compression, mechanical impingement or infection |  |  |
| **Screening/Monitoring** | | | | | |  |
| Testing HLA–B*5801 |  |  |  |  | ✓ (Pt Southeast Asian descent)  O (all other populations) | ✓ (Asian ethnic groups)  O (‘white population’) |
| Cardiovascular risk factors and co-morbid conditions | ✓✓ |  | ✓✓ | ✓✓ |  |  |
| Radiography |  |  |  |  |  | ✓✓ |
| Ultrasound |  |  |  |  |  | ✓✓ |
| Frequency of monitoring |  |  |  |  |  | ? |
| Dual-energy computed tomography |  |  |  |  |  | ? |
| Special populations |  |  |  |  |  |  |
| Solid organ transplant |  |  |  |  |  | ? safest most effective treatment  ✓ Specialist input |

✓✓Should do; ✓ Could do; O Do not do; ? Uncertain.

ACP – American College of Physicians; ACR – American College of Rheumatology; BSR – British Society of Rheumatology; CKD – chronic kidney disease; CV – cardiovascular; e.g. – example EULAR – European League Against Rheumatism; ISR – Italian Society of Rheumatology; IL-1 –interleukin-1; NSAID – non-steroidal anti-inflammatories; SER – Spanish Society of Rheumatology; sUA – serum urate levels; ULT – urate lowering therapy; ; XOI – xanthine oxidase inhibitors.
