# Supplementary material for: What are the core recommendations for gout management in first line and specialist care? Systematic review of clinical practice guidelines
Source: BMC Rheumatol. 2023 Jun 15;7:15. doi: 10.1186/s41927-023-00335-w (PMC10268528; doi:10.1186/s41927-023-00335-w)
Supplement: Supplementary file 4 — Supplementary Material 4. Appendix 4. [file 41927_2023_335_MOESM4_ESM.docx]

**Appendix 4. Description of Clinical Practice Guidelines**

| **Title** | **Year** | **Type of developer** | **Country/Region of Origin** | **Format** | **Informed by CPG quality tool** | **Accompanying documents accessed** | **Guideline topic** | **Target users** |
| --- | --- | --- | --- | --- | --- | --- | --- | --- |
| EULAR [25] | 2016 | Medical Society | Europe | Journal Article | Yes | Lay version  Supplementary Material Document | Non-pharmacological and pharmacological treatments for gout. | Physicians, rheumatologists, GPs, patients and others. |
| ISR [29] | 2019 | Medical Society | Europe | Journal Article | Yes | Nil | Diagnosis and treatment of patients with gout in Italy. | Attending physicians (general practitioners, rheumatologists, internists, nephrologists, cardiologists, geriatricians) and health professionals who manage patients with gout in primary care, and in hospital and community practice settings. Policy makers and those responsible for commissioning care for patients with gout in the Italian National Health Service. |
| ACR [26] | 2020 | Medical Society | USA | Journal Article | Yes | Supplementary Appendix 1-10  Disclosure Summary  Project Plan | Management of gout. | Health care providers and patients with gout. |
| ACP [27] | 2017 | Medical Society | USA | Journal Article | Yes | Full report  Appendix: Detailed methods  Summary of Methods  Systematic review | Management of acute and recurrent gout in adults. | All clinicians. |
| BSR [31] | 2017 | Medical Society | UK | Journal Article | No | Supplementary Data Document | Management of gout in the UK patients. | Doctors and allied health professionals who treat and manage patients with gout in primary care and hospital practice.  Those responsible for commissioning care for patients with gout in the National Health Service. |
| SER [32] | 2020 | Medical society | Europe – Spain | Report | Yes | N/a | Treatment,  diagnosing and monitoring Gout. | Rheumatologists, other health professionals working in primary or specialist care, namely, those from the specialities of cardiology, nephrology, urology, family medicine, and nursing, as well as other specialities potentially involved in the care of these patients and patients and family members. |

ACP - American College of Physicians; ACR - American College of Rheumatology; BSR – British Society of Rheumatology; EULAR - European League Against Rheumatism; GP – General practitioner; ISR – Italian Society of Rheumatology; SER – Spanish Society of Rheumatology; USA – United States of America; UK – United Kingdom.
