# Supplementary material for: What are the core recommendations for gout management in first line and specialist care? Systematic review of clinical practice guidelines
Source: BMC Rheumatol. 2023 Jun 15;7:15. doi: 10.1186/s41927-023-00335-w (PMC10268528; doi:10.1186/s41927-023-00335-w)
Supplement: Supplementary file 5 — Supplementary Material 5. Appendix 5. [file 41927_2023_335_MOESM5_ESM.docx]

**Appendix 5. Overall mean (standard deviation) percentage scores in each AGREE II domain and mean (1-7 scale/standard deviation) for each item.**

|  | **Mean (SD)** |
| --- | --- |
| **Domain 1. Scope and Purpose (%)** | **86% (11.3)** |
| 1. The overall objective(s) of the guideline is (are) specifically described. | 6.6 (0.5) |
| 2. The health question(s) covered by the guideline is (are) specifically described. | 6.0 (1.4) |
| 3. The population (patients, public, etc.) to whom the guideline is meant to apply is specifically described. | 5.8 (1.3) |
| **Domain 2. Stakeholder Involvement (%)** | **79% (15.8)** |
| 4. The guideline development group includes individuals from all relevant professional groups. | 5.8 (0.9) |
| 5. The views and preferences of the target population (patients, public, etc.) have been sought. | 5.1 (1.8) |
| 6. The target users of the guideline are clearly defined. | 6.1 (1.1) |
| **Domain 3. Rigour of Development (%)** | **75% (5.7)** |
| 7. Systematic methods were used to search for evidence. | 6.5 (0.7) |
| 8. The criteria for selecting the evidence are clearly described. | 6.0 (1.5) |
| 9. The strengths and limitations of the body of evidence are clearly described. | 4.9 (1.4) |
| 10. The methods for formulating the recommendations are clearly described. | 5.6 (1.4) |
| 11. The health benefits, side effects, and risks have been considered in formulating the recommendations. | 6.5 (0.7) |
| 12. There is an explicit link between the recommendations and the supporting evidence. | 6.5 (0.9) |
| 13. The guideline has been externally reviewed by experts prior to its publication. | 5.2 (1.1) |
| 14. A procedure for updating the guideline is provided. | 2.7 (2.0) |
| **Domain 4. Clarity of Presentation (%)** | **89% (6.8)** |
| 15. The recommendations are specific and unambiguous. | 6.3 (0.8) |
| 16. The different options for management of the condition or health issue are clearly presented. | 6.2 (1.2) |
| 17. Key recommendations are easily identifiable. | 6.5 (0.9) |
| **Domain 5. Applicability (%)** | **45% (24.8)** |
| 18. The guideline describes facilitators and barriers to its application. | 2.8 (2.3) |
| 19. The guideline provides advice and/or tools on how the recommendations can be put into practice. | 3.8 (2.0) |
| 20. The potential resource implications of applying the recommendations have been considered. | 3.8 (1.9) |
| 21. The guideline presents monitoring and/or auditing criteria. | 4.3 (2.4) |
| **Domain 6. Editorial Independence (%)** | **76% (33.6)** |
| 22. The views of the funding body have not influenced the content of the guideline. | 5.0 (3.0) |
| 23. Competing interests of guideline development group members have been recorded and addressed. | 6.1 (1.2) |
| **Overall Score (%)** | **75% (11.8)** |
