# Supplementary material for: What are the core recommendations for gout management in first line and specialist care? Systematic review of clinical practice guidelines
Source: BMC Rheumatol. 2023 Jun 15;7:15. doi: 10.1186/s41927-023-00335-w (PMC10268528; doi:10.1186/s41927-023-00335-w)
Supplement: Supplementary file 6 — Supplementary Material 6. Appendix 6. [file 41927_2023_335_MOESM6_ESM.docx]

**Appendix 6. CPG AGREE II domain scores and quality assessment (%)**

**Appendix 6.1 CPGs included in the systematic review**

|  | **Doman 1** | **Domain 2** | **Domain 3** | **Domain 4** | **Domain 5** | **Domain 6** | **Overall assessment** | **Domain 2,3,6** |
| --- | --- | --- | --- | --- | --- | --- | --- | --- |
| ACP [27] | 94 | 50 | 74 | 92 | 21 | 100 | 67 | 75 |
| EULAR [25] | 67 | 72 | 70 | 94 | 35 | 42 | 67 | 61 |
| ACR [26] | 81 | 83 | 80 | 92 | 31 | 25 | 67 | 63 |
| ISR [29] | 92 | 83 | 82 | 83 | 48 | 100 | 83 | 89 |
| BSR [31] | 97 | 92 | 74 | 94 | 92 | 100 | 92 | 89 |
| SER [32] | 83 | 92 | 68 | 78 | 40 | 88 | 75 | 82 |

**Appendix 6.2 CPGs excluded from the systematic review**

|  | **Doman 1** | **Domain 2** | **Domain 3** | **Domain 4** | **Domain 5** | **Domain 6** | **Overall assessment** | **Domain 2,3,6** |
| --- | --- | --- | --- | --- | --- | --- | --- | --- |
| *Graf et al. [33] | 42 | 14 | 34 | 64 | 19 | 0 | 33 | 16 |
| APLAR [34] | 81 | 44 | 69 | 78 | 31 | 17 | 53 | 43 |
| FSR – Acute Gout Flare [35] | 53 | 45 | 41 | 67 | 8 | 46 | 50 | 44 |
| FSR – ULT [36] | 47 | 45 | 41 | 67 | 8 | 46 | 42 | 44 |

*First author given where there is no stated organisation; ACP - American College of Physicians; ACR - American College of Rheumatology; APLAR – Asia Pacific League of Associations for Rheumatology; BSR – British Society of Rheumatology; EULAR - European League Against Rheumatism; FSR – French Society of Rheumatology; ISR – Italian Society of Rheumatology; ULT – Urate lowering therapy.
