# Supplementary material for: What are the core recommendations for gout management in first line and specialist care? Systematic review of clinical practice guidelines
Source: BMC Rheumatol. 2023 Jun 15;7:15. doi: 10.1186/s41927-023-00335-w (PMC10268528; doi:10.1186/s41927-023-00335-w)
Supplement: Supplementary file 7 — Supplementary Material 7. Appendix 7. [file 41927_2023_335_MOESM7_ESM.docx]

**Appendix 7. Narrative synthesis of CPG recommendations**

**Appendix 7.1 Gout management interventions narrative summary**

| **Management Interventions** | **Narrative Summary** |
| --- | --- |
| **Non-pharmacological management** | |
| **Education** | Four CPGs strongly [25, 27, 29, 31] and one CPG conditionally [26] recommended providing education for people with gout.  Patients should be supported [29, 31] provided with both verbal and written information [31], including individualised management options, and considering the patients co-morbidities [25, 31], concurrent medications [31] and discussing potential illness perceptions or barriers to care [31]. Content should include education to improve disease knowledge and both non-pharmacological and pharmacological management options. Decision making should be shared between the patient and clinician and be based on a treat-to-target protocol [26].  **Disease knowledge topics**   - The causes and consequences of gout and hyperuricaemia [31]. - Pathophysiology of the disease [25]. - The principles of managing acute attacks and eliminating urate crystals through lifelong lowering of serum urate levels (sUA) level below a target level [25]. - Effective treatments [25].   **Non-pharmacological education topics (diet, exercise, weight management, smoking):**  Lifestyle advice including regarding dietary changes should be provided to all people with gout [25, 31]. Patients should be encouraged to well-balanced diet low in fat and added sugars, and high in vegetables and fibre [31] inclusion of low-fat dairy products [25] such as: skimmed milk and/or low-fat yoghurt alongside, soybeans and vegetable sources of protein, and cherries [31].  CPGs recommended to avoid or limit the following: alcoholic drinks [26, 31] (especially beer and spirits) [25], high- purine foods [25, 26, 31] and sugar-sweetened soft drinks [25, 31], high-fructose corn syrup [26], heavy meals and excessive intake of meat and seafood [25].  Three CPGs strongly encouraged dietary modification to achieve a gradual reduction in body weight and subsequent maintenance in people who are overweight or obese [25, 29, 31]. Whilst one CPG conditionally recommended weight loss for overweight/obese patients with gout, regardless of disease activity [26].  Three CPGs strongly recommended to discuss and encourage regular exercise for all patients with gout [25, 29, 31].  One CPG strongly recommended ceasing smoking in patients with gout [29].    **Pharmacological education topics**  The option of urate lowering therapy (ULT) should be explained to patients following a confirmed diagnosis of gout [31]. Patients with gout should receive full information concerning the use of ULT [25]. Clinicians should explain the rationale [31], aims of its use to target urate levels [31], benefits, harms and costs before initiating ULT [27]. Decision making, including when to commence ULT should consider the individual preferences of the patient [25, 27, 29, 31], with the ULT medication choice being based on the patient factors such as: potential contraindications, past experience of treatments, time of initiation after flare onset and the number and type of joint(s) involved [25].  Clinicians should encourage patients to self-medicate at the onset of symptoms (ideally within 24 hours) during an acute flare [25, 29, 31]. Advice should include continuing any established ULT during and following an attack to prevent the return of symptoms [29, 31].  One CPG strongly recommends gout affected joints during a flare should be rested, elevated, and exposed in a cool environment such as: using ice packs [31]. One CPG conditionally recommends topical ice can be considered [26]. These strategies can be effective as an adjuvant treatment in combination with other therapies [26, 31].  Consensus: Health professions should provide all patients with both written and verbal information including education to improve disease knowledge and both non-pharmacological and pharmacological management options, specifically ULT. Gout affected joints should be rested and elevated.  Ice packs or topical ice could be offered to people with gout. |
| **Vitamin C** | Two CPGs reported conflicting recommendations on vitamin C supplementation for people with Gout. One CPG conditionally recommended for its use at a dosage of 500-1500mg daily due to its weak uricosuric effect [31], whereas one CPG recommended against for all patients with gout, regardless of disease activity [26].  Consensus: No consensus. |
| **Urate-lowering therapies**  One CPG was unable to develop a recommendation for the use of ULT for patients with dialysis due to a lack of evidence [32]. Instead, they recommended that the patient is referred to a unit that has the appropriate clinical experience [32].  Consensus: No consensus. | |
| **Initiating ULT** | Five CPGs strongly recommended initiating ULT in most cases [25, 26, 29, 31, 32], with a xanthine oxidase inhibitor (XOI) as monotherapy [32]. Whilst, one CPG’s recommendations varied on disease severity [26].  Three CPGs strongly recommended that ULT should be discussed and offered to all patients who have a diagnosis of gout at their first presentation [25, 29, 31] and can be considered during a flare [26]. Conversely, one CPG suggested delaying commencement until the inflammation has settled; as they believed ULT is better discussed when the patient is not in pain [31].  CPGs provided criteria for when to initiate ULT:  ULT should be recommended particularly for patients with:   - Recurring attacks [25, 29] (defined as: 52 attacks in 12 months [31] or >2/year [26]). - Radiographic damage (any modality) attributable to gout [26]. - Tophi [25, 26, 29, 31]. - Chronic gouty arthritis [31]. - Joint damage [31]. - Renal impairment (estimated Glomerular Filtration Rate <60ml/min) [31]. - A history of urolithiasis [31]. - Diuretic therapy use [31]. - Primary gout starting at a young age [31] defined as <40 years [25]. - Urate arthropathy and/or renal stones [25, 29] - People with a very high sUA level (>8.0 mg/dl; 480 μmol/L) and/or comorbidities (e.g. renal impairment, hypertension, ischemic heart disease, heart failure) [25, 29].   ULT can be recommended:   - For patients who have previously experienced >1 flare but have infrequent flares (<2/year) or for patients experiencing their first flare and CKD stage >3, sUA >9 mg/dl, or urolithiasis [26].   ULT should not be recommended for:   - For patients experiencing their first flare or patients with asymptomatic hyperuricemia (sUA >6.8 mg/dl with no prior gout flares or subcutaneous tophi) [26].   Consensus: No consensus. |
| **Aim and monitoring sUA level** | Two CPGs strongly recommended monitoring sUA level [25, 29], frequency of gout attacks and tophi size [29]. Four CPGs strongly recommended maintaining a low target sUA [26, 31, 32] for faster crystal elimination [26, 31]; with the treatment target being an absence of gout attacks and resolution of tophi [29].  sUA target levels vary depending on gout severity. sUA <5 mg/dL (300 mmol/L) is recommended for patients with severe gout (defined by - tophi, chronic arthropathy, frequent attacks) [25, 29, 32], or initial gout episode [31] as a lower sUA target can assist with crystal dissolution [25, 31] and prevent further urate crystal formation [31].  Clinicians can consider increasing the sUA target to <6 mg/dL (360 mmol/L) [25, 26, 29, 31] when the patient has experienced several years of successful treatment, tophi has resolved, and the patient is symptom free [31]. This should be monitored and maintained lifelong [25, 26, 29, 31]. The same target sUA levels should be used for patients who have chronic kidney disease [32].  A sUA level <3 mg/dL is not recommended long-term [29], due to the possibility of adverse effects [29, 31].  Consensus: sUA level should be monitored and maintained to <6 mg/dL (360 mmol/L) lifelong, or sUA <5 mg/dL (300 mmol/L) for patients with severe gout or those experiencing an initial gout episode.  A sUA level <3 mg/dL is not recommended in the long term. |
| **Dosage of ULT** | Three CPGs strongly recommended delivery of ULT following a treat-to-target strategy [26], that includes starting from a low dose [25, 32] and dose titration [25, 26, 32] upwards to achieve the sUA target [25, 26, 32] of <6 mg/dL (360 mmol/L) [25].  This is preferred over a fixed, standard-dose ULT strategy [26].  Consensus: ULT dosage should be selected based on a target-to-treat strategy and titrated upwards to achieve a sUA target of <6 mg/dL (360 mmol/L) long-term. |
| **Length of ULT therapy** | One CPG conditionally recommended continuing ULT indefinitely over stopping ULT [26] whereas one CPG conditionally recommended for ULT in shorter durations, however they recommend against long-term (≥12 months) ULT use in majority of patients after a first gout attack or in patients with infrequent attacks due to lack of evidence [27].  Consensus: No consensus. |
| **Acute treatment** | |
| **NSAIDs** | All CPGs strongly recommended non-steroidal anti-inflammatories (NSAIDs) [25-27, 29, 31, 32] with two CPGs recommending against for people with gout and renal impairment [25, 29].  Initial combination therapy is an appropriate option for acute [27] gout attack [29] when there are no contraindications [31] and when monotherapy is insufficient [29, 31].  NSAID (+/-plus proton pump inhibitors)[25, 31] at maximum dose [31] should be considered as a first-line treatment option, alongside colchicine [25, 26, 29, 31] or corticosteroids [25-27, 29]. These medications are effective in reducing pain [27], and should be discussed with the patient. Treatment decisions should consider patient factors [26] including patient preference [31, 32], presence of comorbidities [29, 31, 32] e.g. impaired renal function, contraindications, the number and type of joint(s) involved [29].  NSAIDs should be avoided in patients with severe renal impairment who are experiencing an acute gouty attacks [25, 29].  Consensus: NSAIDs should be recommended for people with gout as a first-line treatment, taking into consideration patient factors.  NSAIDs should not be recommended for people with gout and renal impairment. |
| **Colchicine** | All CPGs strongly recommended colchicine [25-27, 29, 31] as the drug of choice (alongside NSAIDs or corticosteroids) for managing an acute gouty attack when there are no contraindications [31]; avoid for patients with renal impairment [25, 29, 31] or for patients who are receiving strong P-glycoprotein and/or CYP3A4 inhibitors such as cyclosporin or clarithromycin [25].  This should be initiated within 12 hours of flare onset [25], at a low-dose [26, 27]. Dosage varied from 1.2 mg followed by 0.6 mg 1 h later [27] to 1 mg followed 1 hour later by 0.5 mg [25] or 500mg bd-qds [31]. Low doses are seen as effective as higher dosages for reducing pain and are associated with fewer gastrointestinal adverse effects [27]. In patients who cannot tolerate colchicine, a low-dose NSAID or coxib, with gastroprotection can be used as an alternative providing there are no contraindications [31].  These options should be discussed with the patient and treatment decisions should consider patient factors [26] including patient preference [31], presence of comorbidities [29, 31] e.g. impaired renal function, contraindications, the number and type of joint(s) involved [29]. Initial combination therapy is an appropriate option for a severe gouty attack [29].  Consensus: Colchicine should be recommended for people with gout as a first-line treatment, taking into consideration patient factors.  Colchicine should not be recommended for people with gout and renal impairment or who are receiving strong P-glycoprotein and/or CYP3A4 inhibitors. |
| **Corticosteroids** | Five CPGs strongly recommended for the use of corticosteroids [25-27, 29, 32], whilst one conditionally recommended for this intervention [31] as a first-line option for an acute gout flare alongside NSAIDs, colchicine [27, 29, 32]; with the choice of treatment depending on patient preferences and comorbidities [32].  For patients who are unable to tolerate NSAIDs or colchicine, a short course of oral corticosteroid [31] or joint aspiration [31] and a single injection of an intramuscular corticosteroid [31] [26, 29], intra-articular or intravenous [26, 29] can be considered.  Corticosteroids either an intramuscular injection or short-course orally is considered appropriate for oligo- or polyarticular attacks of gout [31]. Corticosteroids and corticotrophin [29] are recommended over IL-1 inhibitors or adrenocorticotropic hormone [26].  Consensus: Corticosteroids should be offered to patients with acute gout. |
| **Prevention** | |
| **Prophylaxis** | Three CPGs strongly recommended for the use of prophylaxis for people with gout [25, 26, 29]. Prophylaxis should be fully explained and discussed with the patient [25]. Treatment should be initiated prior to or within the first 6 months of ULT [25, 29] or upon resolution of acute gout [26] and continued for 3–6 months, with ongoing evaluation and continued prophylaxis as needed if the patient continues to experience flare [26]. A dosage of 0.5–1 mg/day is recommended, that should be reduced in patients with renal impairment [25].  Colchicine is recommended as the first choice of prophylactic treatment, followed by a low-dose NSAID [25, 26, 29] or COXIB [25, 29] and lastly low-dose glucocorticoids if the other options are contraindicated, not tolerated, or ineffective [29].  Alternatively to low-dose colchicine or NSAIDs, febuxostat (40 mg/d) and allopurinol (300 mg/d) are equally effective at decreasing sUA levels [26].  Neurotoxicity and/or muscular toxicity risk should be considered, if a patient has renal impairment or is receiving statin treatment [25]. Avoid co- prescription of colchicine with strong P-glycoprotein and/or CYP3A4 inhibitors [25].  Consensus: Prophylaxis should be initiated prior to or within the first 6 months of ULT and continued for at least 3-6 months at a dosage of 0.5-1mg/day. Lower dosage and side effects of colchicine should be considered for people with renal impairment. |
| **XOI** | Two CPGs strongly recommended the use of an XOI (allopurinol or febuxostat) for people with chronic kidney disease (CKD) [26, 32] stage >3 [26] as a first-line treatment [26, 32] over probenecid [26] with specific limitations stated in their summary of product characteristics [32].  One CPG recommended that for patients who are taking their first XOI monotherapy at a maximum dose, and who are not achieving target sUA and/or continue to have frequent flares or non-resolving subcutaneous tophi, consider switching to an alternate XOI agent instead of a uricosuric agent [26]. Whereas the other CPG recommended to combine XOI with a uricosuric agent [32].  Consensus: XOI should be recommended for patients with CKD. |
| **Allopurinol** | **Allopurinol**  Five CPGs strongly recommended the use of allopurinol as a first-line ULT [25, 26, 29, 31, 32] for people with gout and with normal kidney function [25], including in those with CKD [26, 32] stage >3 [26].  It is recommended to start at a low dose of 50-100mg daily [31] (no greater than 100mg) [25, 26, 29] and lower in patients with CKD [26]. Dosage should be increased by 100mg increments [25, 31] every 2-4 weeks [25] or 4 weeks [31] until sUA target is reached [25, 29, 31, 32], with a maximum dose of 900mg [31].  In patients with renal impairment (any grade), allopurinol may be used, if the initial dosage starts at 50 to 100 mg daily for the lowest levels of renal function [32]. Doses should be gradually increased by smaller increments (50mg [29, 31, 32] – 100mg depending on renal function [32]) and the maximum dose will be lower [31]. Dose should be adjusted to creatinine clearance [25], but target sUA levels should be the same [31]. Clinicians should conduct close monitoring for adverse events and toxicity (e.g., pruritus, rash, elevated hepatic transaminases [29].  If the sUA target cannot be achieved [29] or allopurinol cannot be tolerated, febuxostat [25, 29, 32] or benzbromarone with or without allopurinol can be considered except for patients with estimated glomerular filtration rate <30 mL/min [25].  Febuxostat can be given at a dosage of 40mg/d [26] and/or a uricosuric, can be considered [25, 29]. For patients with a prior allergic response to allopurinol who cannot be treated with other oral ULT, we conditionally recommend using allopurinol desensitization [26].  One CPG recommended that allopurinol should be avoided in patients who are known to have the HLA-B*58 allele (such as those who are transplant recipients or on a transplant programme) [32].  Consensus: Allopurinol should be recommended as a first line ULT at 50-100mg daily and increased 100mg increments every 2-4 weeks until sUA target is reached with a maximum dose of 900mg.  Consider febuxostat and/or uricosuric if allopurinol is not effective or contraindicated.  Allopurinol should be avoided for patients with HLA-B*58 allele |
| **Febuxostat** | Two CPGs conditionally recommended [29, 31], one CPG strongly recommended [26] the use of febuxostat as an alternative second-line XOI where allopurinol has not been effective in achieving the therapeutic sUA target [29, 31] or if renal impairment prevents allopurinol dose escalation [31]. It can be considered as a first-line therapy if a patient has severe gout and requires a extremely low sUA target [32].  CPGs recommended starting at a low dose with subsequent dose titration (e.g. <100mg/day for patients without CKD and lower for patients with CKD [26], whilst the other suggested 80mg daily [31]. One CPG recommended increasing the dosage after 4 weeks to 120 mg daily, if necessary to achieve therapeutic target [31].    Febuxostat can be considered for patients with gout and CKD [32]. Patients with a high cardiovascular (CV) risk but no history of a CV event should consider the benefits/risks of this medication before potentially commencing [32]. If they have a history of cardiovascular disease (CVD) or have experienced a new CV event, consider switching to an alternative ULT agent if available and consistent with other recommendations in this guideline [26].  Consensus: Febuxostat can be considered for patients who have renal impairment and allopurinol is either contraindicated or hasn’t allowed for therapeutic sUA target to be achieved.  Clinicians can consider other ULT agents for patients who have a history of CVD or a new CV event. |
| **Uricosuric agents** | Two CPGs conditionally recommended and one CPG strongly recommended for the use of uricosuric agents either in combination with a XOI in patients who do not achieve a therapeutic serum urate target [29, 31, 32] with optimal doses of monotherapy XOI [31, 32] (in appropriate doses or maximum tolerated doses [32]) or in replacement of XOI’s in patients who are resistant to, or intolerant of, XOIs [31] and have severe gout [29].  The preferred uricosuric agents are:   - Sulfinpyrazone (200-800 mg/day) [31]. - Probenecid (500-2000 mg/ day) in patients with normal or mildly impaired renal function [31]. - Benzbromarone (50-200 mg/ day) in patients with mild to moderate renal insufficiency [31].   - Benzbromarone can be considered as monotherapy if a patient has a poor response to treatment, are intolerant or has an adverse reaction to XOIs. Similarly, monotherapy can be considered if they have CVD or a history of CV event [32].   - If a patient has CKD, benzbromarone should only be prescribed after a poor response or adverse effects related to an XOI [32]. - Lesinurad only in combination with XOIs and should be considered before benzbromarone [32]. Taking into   account the warnings and precautions for its use [32].  Lesinurad or benzbromarone should not be prescribed for patients with severe kidney disease [32]. Avoid Lesinurad if a patient has experienced a vascular event in the last 12 months [32]. One CPG recommended against checking urinary uric acid and alkalinizing urine for people taking uricosuric treatment [26].  Consensus: Uricosuric agents can be considered either in combination with XOI or as monotherapy in patients who have a poor response, are intolerant or have an adverse reaction to XOI’s.  Lesinurad or benzbromarone should not be prescribed for patients with severe kidney disease.  Checking of urinary uric acid and alkalinizing urine for people taking uricosuric treatment is recommended against. |
| **Pegloticase** | One CPG conditionally recommended for the use of pegloticase for people with gout in particular cases [25]. Whilst two CPG reports conflicting recommendations, they strongly recommended for in some cases and against in other cases [26] or reported a combination of strongly and conditionally recommended for pegloticase [32].  Pegloticase is not recommended as a first-line therapy or for patients currently continuing ULT for patients who have failed to achieve sUA target with other treatment interventions such as XOIs, uricosurics, but who have infrequent gout flares (<2 flares/year) and no tophi [26].  Conversely, for patients who have failed to achieve sUA target [25, 26, 32] with other treatment interventions [26] and who have crystal-proven, sever debilitating chronic tophaceous gout and poor quality of life [25], or frequent gout flares (≥2 flares/year), or who have non-resolving subcutaneous tophi [26], pegloticase could [25] or should [26] be used.  Pegloticase should be considered for patients with severe kidney disease, who haven’t reached sUA target and who haven’t tolerated other treatment options or as an alternative option to lesinurad + XOI, or benzbromarone as monotherapy in those who have a history of a CV event [32].  Consensus: No consensus. |
| **Fenofibrate +/- Losartan** | One CPG recommended against adding or switching to fenofibrate for people with gout [26]. One CPG recommended against the use of losartan and fenofibrate as primary ULT, however conditionally recommended for their combined use in treating hypertension or dyslipidaemia as they can have a weak uricosuric effect [31].  Consensus: No consensus. |
| **IL-1 inhibitors** | Four CPGs conditionally recommended for the use of interleukin 1 (IL-1) inhibitors [25, 26, 31] for people with acute gout [31] or who have frequent flares [25] and contraindications [25, 26, 29] or haven’t responded adequately to standard treatment [26, 29, 31] such as: colchicine, NSAIDs and corticosteroid (oral and injectable)[25].  IL-1 inhibitors should not be considered if the patient has a current infection. ULT should be adjusted to achieve the sUA target following an IL-1 blocker treatment for flare [25].  Consensus: IL-1 inhibitors could be considered for people with acute gout, and/or frequent flares who have contraindications or haven’t responded adequately to standard treatment.  Do not use IL-1 inhibitors if the patient has a current infection. |
| **Antihypertensive medications** | Three CPGs conditionally recommended anti-hypertensive medications in certain circumstances [25, 26, 31]. Two CPGs conditionally recommend switching from hydrochlorothiazide to an alternate antihypertensive [26], preferably losartan [25, 26] or calcium channel blockers [25] to treat hypertension, when blood pressure is controlled [31] regardless of disease activity [26].  If a patient has gout and hyperlipidaemia and is receiving loop or thiazide diuretics, consider replacing the diuretics for a statin or fenofibrate [25].  Consensus: People with gout and hypertension could consider switching from diuretic drugs to an alternate antihypertensive medication preferably losartan or calcium channel blockers.  People with gout and hyperlipidaemia could consider switching from diuretic drugs to an alternate antihypertensive medication statin or fenofibrate. |
| **Screening/Monitoring** | |
| **Cardiovascular risk factors and co-morbid conditions** | Three CPGs strongly recommended screening for cardiovascular risk factors, renal function and co-morbid conditions in all patients with gout [25, 29, 31] at the time of diagnosis [29], reviewed annually and managed appropriately [29, 31].  Cardiovascular risk factors, include: renal impairment [25, 29, 31], coronary heart disease [25], heart failure [25], stroke, [25], peripheral arterial disease [25], obesity, hyperlipidaemia, hypertension, diabetes mellitus, and smoking [25, 29, 31].  Consensus: All patients should be screened for cardiovascular risk factors and co-morbid conditions initially and reviewed annually. |
| **Testing HLA–B*5801** | Two CPGs conditionally recommended for testing HLA–5801 prior to starting allopurinol for patients of Southeast Asian descent (e.g., Han Chinese, Korean, Thai) [26, 32] and African American patients, who have a higher prevalence of HLA–B*580 [26]. Both CPGs recommended against testing in all other population groups [26, 32].  Consensus: Southeast Asian descent and African American patients can be tested for HLA–5801 prior to starting allopurinol.  Testing in other populations groups is recommended against. |

CKD – chronic kidney disease; COXIB – cyclooxygenase-2 inhibitors; CPGs – Clinical Practice Guidelines; CV – cardiovascular; CVD – cardiovascular disease; IL-1 – Interleukin-1; NSAID – non-steroidal anti-inflammatories; sUA – serum uric acid; ULT – urate lowering therapy; XOI – xanthine oxidase inhibitor.

**Appendix 7.2 Single CPG recommendations on interventions**

| **Non-pharmacological** |
| --- |
| **Adequate water intake**  One CPG strongly recommended >2 litres of water daily and avoid dehydration for people with gout and a history of urolithiasis. Alkalinization of the urine with potassium citrate (60 milliequivalent/day) should be considered in recurrent stone formers [31].  **Nurses involvement**  One CPG strongly recommended for the support and regular follow-up of a specially-trained nurse for patients with gout [32]**.** |
| **Prevention** |
| **Probenecid**  One CPG conditionally recommended for the use of probenecid, starting at a low dose (500 mg once or twice daily) with dose titration over starting at a higher dose [26].  **Stopping aspirin**  One CPG recommended against stopping low-dose aspirin in those with gout who are taking this medication for appropriate indications [26].  **Two medications with the same mechanism of action**  One CPG recommended against two medications with the same mechanism of action (e.g. two XOI’s) due to a lack of evidence. |
| **Management options** |
| **Medical vs Surgical**  One CPG strongly recommended medical management to achieve a sustained reduction in sUA, only considering surgical management for selected cases such as: nerve compression, mechanical impingement, or infection [29]. |
| **Screening/Monitoring** |
| **Radiography**  One CPG recommended that plain radiography is used to assess the extent of joint damage and monitor bone erosions [32].  **Ultrasound**  One CPG recommended that ultrasound is used to assess the effect of ULT, checking for urate deposits, double-contour sign and size of tophi [32].  **Frequency of monitoring**  One CPG was unable to determine a optimal time interval for examinations [32].  **Dual energy computed tomography**  One CPG was unable to recommend either for or against the use of dual energy computed tomography for follow-up [32]. |
| **Special populations** |
| **Solid organ transplant**  One CPG was unable to determine the most effective and safest treatment option for people with gout who have received a solid organ transplant [32].  These patients can have specialist input from nephrology, hepatology, and rheumatology units with experience in these cases [32]. |

CPG – clinical practice guideline; sUA – serum uric acid; ULT – urate lowering therapy; XOI – xanthine oxidase inhibitor.
